# The influence of mobile phone-based health reminders on patient adherence to medications and healthy lifestyle recommendations for effective management of diabetes type 2: a randomized control trial in Dhaka, Bangladesh

**DOI:** 10.1186/s12913-020-05387-z

**Published:** 2020-06-08

**Authors:** Farzana Yasmin, Nazmun Nahar, Bilkis Banu, Liaquat Ali, Rainer Sauerborn, Aurélia Souares

**Affiliations:** 1 Heidelberg Institute of Global Health (HIGH), Heidelberg University Hospital, Heidelberg, Germany; 2 Friede Springer Endowed Professorship for Global Child Health, Faculty of Health/Department of Human Medicine, University of Witten/Herdecke, Alfred-Herrhausen-Str. 50, 58448 Witten, Germany; 3 Independent Consultant, Marburg, Germany; 4 Pothikrit Institute of Health Studies, Dhaka, Bangladesh

**Keywords:** Adherence, Diabetes type 2, Mobile health, Health outcomes, Interactive voice call, Call center, Bangladesh

## Abstract

**Background:**

In 2017, 80% of 425 million adults with diabetes worldwide were living in low and middle-income countries. Diabetes affected 6.9 million adults in Bangladesh and accounted for 3% of the country’s total mortality. Proper management of diabetes is the key to positive health outcomes. This study investigated how mobile phone-based health intervention could increase patient adherence and thereby improve the disease outcomes for diabetes type 2 in Bangladesh.

**Methods:**

A mobile phone-based health project (including mobile phone reminders and 24/7 call center) was implemented in Dhaka District, Bangladesh from January to December 2014. A randomized control trial was carried out, recruiting randomly in intervention and control groups among the patients receiving treatment for type 2 diabetes at the Bangladesh Institute of Health Sciences Hospital, Dhaka, Bangladesh. A total of 320 patients from both groups at baseline and 273 at endline were interviewed.

**Results:**

A significant improvement in patient adherence to diet, physical exercise, the cessation of use of tobacco and betel nut, and blood glycaemic control was found in the intervention group, whereas no such significant improvement was found in the control group. Cost and other co-morbidities were found to be the main reasons for non-adherence.

**Conclusion:**

A mobile-health intervention should be considered as an additional option for non-communicable disease programs.

## Background

Non-communicable diseases (NCDs) have emerged as a serious challenge for health and economic development in low and middle-income countries (LMICs) [1]. NCDs have considerably increased premature mortality and morbidity and put a double burden of diseases (adding to existing burden of communicable diseases) on health systems through increased service utilization and overall treatment cost [1, 2]. In resource-limited health settings, NCDs also act as a barrier to poverty alleviation and sustainable development [1, 2]. Among the NCDs, diabetes is one of the rising public health concerns for both developed and developing countries [1]. In 2017, 425 million adults (20–79) worldwide had diabetes, 80% of them were living in LMICs. The number is projected to increase up to 629 million by 2045 [2]. Diabetes-related deaths were four million worldwide in 2017 [2]. In the same year, Bangladesh had 6.9 million adults living with diabetes; the number is projected to increase up to 13.7 million by 2045 [2, 3]. Diabetes-related deaths account for 3% of the total mortality in the country [4]. In 2010, diabetes ranked 16th for the years of life lost (YLL) and 12th for the disability-adjusted life years (DALY) in Bangladesh [5, 6]. The annual cost of diabetes is USD 51.4 per person in Bangladesh; up to 40% of the people could not afford treatment in 2011 [3, 7] mainly due to the high percentage (67%) of out-of-pocket payment (OOP) [8]. Bangladesh is now experiencing a demographic and epidemiological transition. Rapid urban growth (3.5% annually) has led to increased sedentary lifestyles, higher calorie consumption, and more stressful life conditions. All these elements combined with a growing elderly population and lack of awareness about healthy lifestyles contributed to the increased diabetes prevalence [9, 10].

Adherence to treatment for chronic diseases such as diabetes is crucial for better outcomes. Adherence is defined as the extent to which a patient’s behavior corresponds with the agreed medications and lifestyle recommendations from a health care provider [11]. Non-adherence evidenced higher mortality and morbidity, progression of complications, poor disease outcomes, and an overall lower quality of life [12]. It has also economic consequences including repeated physician visit and laboratory test, increase hospitalization, disability, and premature death [13, 14]. Adherence varies widely across different disease conditions, treatment regimens, and patient populations. On average, 25% patients are non-adherent to acute diseases treatment, whereas 50% or more in chronic conditions such as diabetes [13, 15, 16]. The key steps to delay disease progression are early diagnosis, access and adherence to treatment, and adherence to healthy lifestyle modification recommendations, particularly a healthy diet and physical exercise practice. Therefore, mobile health or m-Health (mobile phone-based delivery of health services) came into consideration as an addition to the existing health system to ensure the sustainable and cost-effective treatment of DM 2 not challenging further the country’s health system and economy and to improve patients’ adherence and thereby, disease outcomes.

In Bangladesh, 97.5% (156 million people out of 160 million) of individuals had access to a mobile phone in 2018 [17, 18]. This wide range of coverage offers the opportunity to use mobile phone-based services (such as short message services SMS, interactive voice calls, call centers etc.) to improve the provision of personalized health care. This could potentially enhance adherence and disease outcomes, which in turn may help to reduce health system burden and health care cost in LMICs [19]. But, the utilization of m-Health services has not yet reached its full potential. This is especially the case for the following sub-groups: the elderly, who lack both information on modern technology and motivation to use it (factor age); female, who have less ownership as a result of male control over household mobile phone ownership (factor sex); illiterate people, who have less access, information, use, acceptance and understanding of modern technology (factor literacy); low income group, who lack money to top-up a mobile phone (factor economic status); and population in rural and hard-to-reach areas, who lack a mobile network, infrastructure and electricity (factor geographical location) [20–30]. But, no study has been conducted in Bangladesh so far using only mobile phone interactive voice call applications independent of internet access to increase the patients, adherence and disease outcomes of diabetes type 2 (DM 2) irrespective of their socio-demographic and economic status. Therefore, this study hypothesized that mobile phone-based health reminders increase patient adherence to medications and other healthy lifestyle modification recommendations for effective management of DM 2 in Bangladesh.

## Methods

### Study design, sampling and data collection

**Fig. 1:**
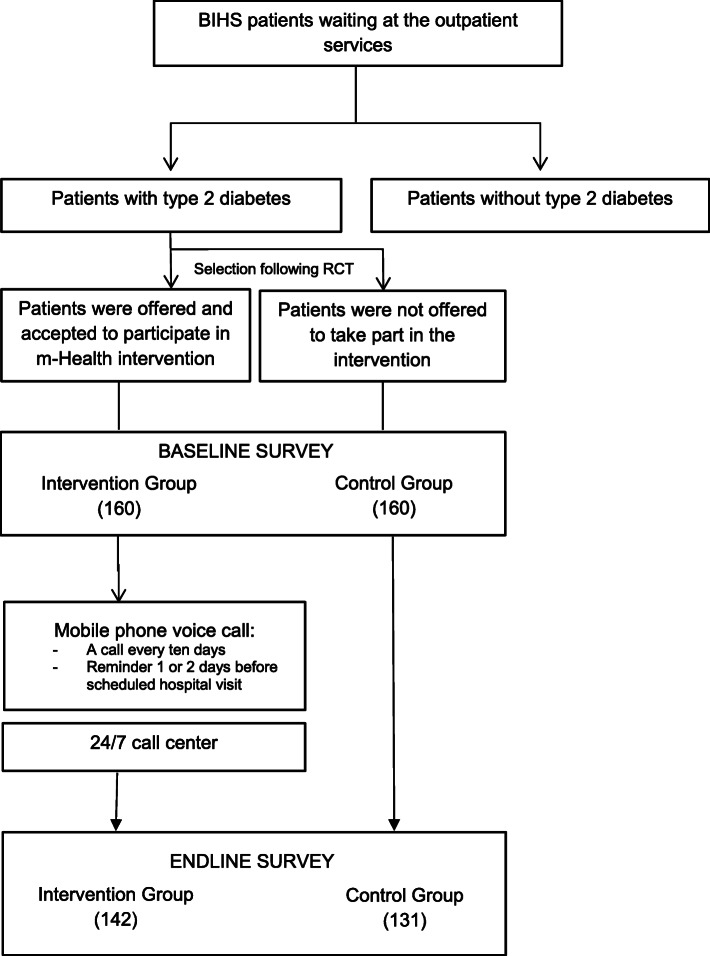
Flow chart of the study and services offered under m-Health project

A mixed-method, sequential explanatory design was used in this study. The quantitative part was followed by a smaller qualitative component (18 qualitative in-depth interviews have been conducted among the patients included in the survey). Only the quantitative results will be presented in this paper. An m-Health intervention was implemented for one year (January to December 2014) in Dhaka district, Bangladesh. It was a joint study of the Heidelberg Institute of Global Health (HIGH), Heidelberg University Hospital, Germany and the Bangladesh University of Health Sciences (BUHS), Bangladesh. Patients who visited the out-patient department of the Bangladesh Institute of Health Sciences (BIHS) hospital, Dhaka for DM 2 consultation constituted the sampling frame. The sample size was calculated based on an expected 15% increase in adherence, with *p* = 0.05 and 80% power. The expected increase of adherence was taken based on the local analysis of experts (BUHS research group). Based on the calculation, 320 patients were included in the baseline study with an equal number of patients in the intervention (160) and in the control group (160) following a non blinded Randomized Control Trial (RCT) design (Fig. 1). The concept of the intervention was explained to the participants of both groups and they were informed in which arm they were included. The inclusion in the intervention or control group was only done upon participants agreement.

**Fig. 2:**
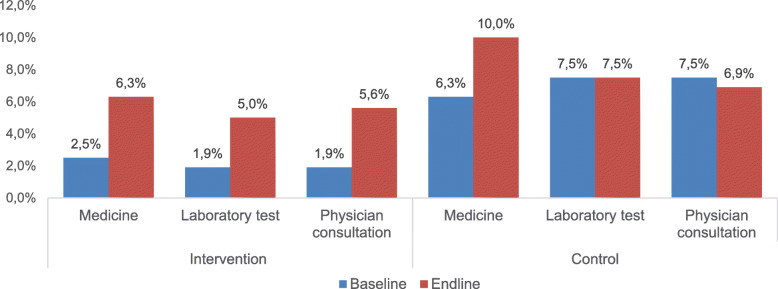
Percentage of patients omitting medicine intake and hospital visits (physician consultation and laboratory test) due to cost. The patients in both groups omitted medicines, physician consultation and laboratory tests due to cost. This figure shows that the percentage has increased for medicine intake, laboratory test and physician consultation in the intervention group between baseline and endline, but it has only increased slightly in medicine intake, stayed similar for laboratory test and decreased slightly for physician consultation in the control group

**Table 1:** Socio-demographic characteristics of the intervention and the control group

Variables	Intervention		Control		*p* value
Group Size	*N* = 160	100.0%	*N* = 160	100.0%	
Average Age [range in years]	53 [30–85]		51 [30–75]		0.011
No. of female	119	74.0%	130	81.0%	0.14
Marital status					0.422
Never married	0	0.0%	1	0.5%	
Married	126	79.0%	130	81.0%	
Divorced	1	0.5%	2	1.5%	
Widow	33	20.5%	27	17.0%	
Education					0.017
No Formal schooling	24	15.0%	27	17.0%	
Primary school	39	25.0%	44	28.0%	
Secondary school	83	52.0%	89	55.0%	
University level	14	9.0%	0	0.0%	
Work status					0.524
Government Job	9	5.5%	9	5.5%	
Private Company	3	2.0%	12	8.0%	
Self-employed (business)	21	13.0%	14	9.0%	
Homemaker (housewife)	110	69.0%	114	71.0%	
Retired person	17	10.5%	10	6.0%	
Unable to work	0	0.0%	1	0.5%	
Type of family					1.000
Nuclear	120	75.0%	120	75.0%	
Combined	40	25.0%	40	25.0%	
No. of family members contributing in family income	116				0.142
1 member	34	73.0%	102	64.0%	
2 members	7	21.0%	44	27.0%	
3 members	3	4.0%	11	7.0%	
4 members		2.0%	3	2.0%	
Monthly Family Income (in Euro; 1 Euro = 86.024 BDT)					0.233
< 116	12	7.0%	19	12.0%	
117–465	88	55.0%	91	56.0%	
> 465	40	25.0%	36	22.5%	
Refused to answer	3	1.5%	7	4.0%	
Don’t know	17	11.5%	7	4.0%

**Fig. 3:**
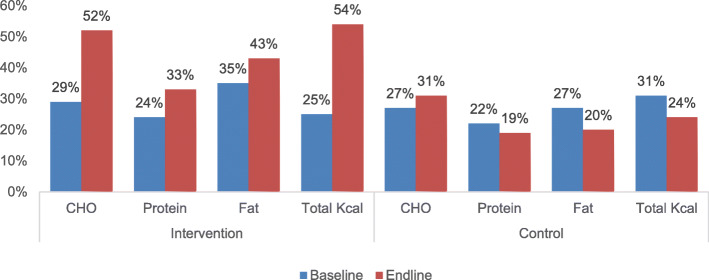
Adherence to calorie intake (CHO, protein, fat and total kilocalorie intake). This figure shows the percentage of adherence to calorie intake in both the intervention and the control groups. The adherence to CHO has almost doubled and the adherence to total kilocalorie has more than doubled in the intervention group between baseline and endline; the increase is not significant for protein and fat intake. In the control group, the adherence to CHO intake has increased slightly but decreased slightly for protein, fat and total kilocalorie intake between baseline and endline

**Table 2:** Mean number of days of exercise per week and hours of exercise per day

Exercise-days per week	Intervention		Control	
	Baseline	Endline	Baseline	Endline
Mean	4.6	6.13	5.08	5.28
*p* value	*p* = < 0.001		*p* = 0.538	
**Exercise-hours per day**	**Intervention**		**Control**	
	Baseline	Endline	Baseline	Endline
Mean	0.45	0.52	0.57	0.45
*p* value	*p* = 0.118		*p* = 0.027

**Table 3:** Bio-Medical information at last visit

Variable		Intervention		Control	
		Baseline	Endline	Baseline	Endline
**Fasting blood glucose level (ref. value < 7.0 mmol/L)**	Mean	8.67	6.80	8.54	8.13
	*p* value	*p* = < 0.001		*p* = 0.256	
**Blood glucose 2 h after breakfast** **(ref. value < 11.1mmom/L)**	Mean	12.33	9.84	12.57	11.67
	*p* value	*p* = < 0.001		*p* = 0.035

**Table 4:** Factors associated with adherence to total calorie intake, physical exercise, betel nut and smokeless tobacco cessation, and fasting blood glucose control

Variables		Adherence: total calorie intake					
		crude OR	95% CI	p	adjusted OR	95% CI	p
Group	Intervention	3.82	2.26, 6.43	0.00	4.00	2.34, 6.83	0.00
Sex	Male	1.98	1.13, 3.48	0.017	1.87	1.02, 3.41	0.04
Adherence at baseline	Adherent	1.43	0.84, 2.42	0.18	1.62	0.91, 2.88	0.09
		Adherence: walking 5 or more days in a week					
		crude OR	95% CI	p	adjusted OR	95% CI	p
Group	Intervention	2.37	1.24, 4.52	0.008	3.07	1.52, 6.19	0.002
Sex	Male	0.57	0.29, 1.13	0.11	0.72	0.34, 1.52	0.39
Age	25–40 years	ref.			ref.		
	41–60 years	0.49	0.14, 1.71	0.26	0.40	0.11, 1.47	0.17
	> 60 years	0.12	0.03, 0.45	0.002	0.10	0.02, 0.41	0.001
Adherence at baseline	Adherent	1.91	1.01, 3.62	0.045	2.37	1.18, 4.77	0.01
		Adherence: did not smoke tobacco					
		crude OR	95% CI	p	adjusted OR	95% CI	p
Group	Intervention	5.11	2.54, 10.25	0.00	5.75	2.70,12.21	0.00
Sex	Male	2.41	1.03, 5.65	0.041	1.36	0.59, 4.24	0.52
Education	Up to primary level	ref.			ref.		
	Up to higher secondary	2.62	1.41, 4.85	0.002	2.42	1.20, 4.86	0.01
	Graduation and above	5.59	0.70, 44.67	0.10	1.34	0.14,12.12	0.79
Adherence at baseline	Adherent	2.79	1.48, 5.23	0.001	2.38	1.18, 4.81	0.01
		Fasting blood glucose control (controlled level = < 7.0 mmol/L)					
		crude OR	95% CI	p	adjusted OR	95% CI	p
Group	Intervention	2.97	1.78, 4.93	0.00	3.1	1.81, 5.3	0.00
Sex	Male	0.97	1.23, 1.73	0.94	1.0	0.53, 5.3	0.98
Cost total medicine	> 3000 taka	0.41	0.25, 0.69	0.001	0.46	0.27, 0.79	0.005
Fasting blood glucose control at baseline		2.68	1.59, 4.50	0.00	2.97	1.71, 5.16	0.00

According to hospital data, around 40 patients were receiving health services for DM 2 at the BIHS out-patient department daily. Considering the time frame of data collection (2 months or 50 working days) and the need to achieve the total calculated sample size, seven interviews were conducted each day. The first patient was randomly selected for each group (intervention and control) using the lottery method among the first five patients by the interviewer. From then on, every fifth patient was interviewed for both groups. In case a patient did not agree to participate or was not fulfilling the inclusion criteria (receiving treatment for DM 2, having a personal mobile phone and voluntary participation), the next patient was selected. Each patient of the intervention group signed an informed consent form both at baseline and endline surveys. Patients were interviewed immediately after getting enrolled and registered into the electronic server system. An automated message was sent to every patient’s mobile phone informing them about their integration into the system and the opportunity to call for the 24/7 call center services. Due to the overall low literacy rate and the presumed lower ability to use all functions of a mobile phone (especially by the older age group), each patient also received a personal call to confirm his/her registration. It also served the purpose to validate the patient provided number. After validation, 17 patients were replaced following the same procedure due to an incorrect number or a switched off mobile phone. During baseline data collection, two patients from the intervention and three patients from the control group were unable to take part in the interview due to sickness and were replaced. No patient refused to take part in the interview in general or to be included in intervention or control group. The baseline data collection period was January to February 2014.

The endline data collection period was extended from January up to May 2015 due to political unrest in the country. A structured questionnaire was used to conduct the baseline and the endline survey. The questionnaire enclosed 12 sub-sections: (1) General survey information including interview ID, groups, name, address, mobile number, date and time of the interview, (2) Socio-demographic information, (3) Diseases and therapeutic information, (4) Knowledge and perception of diabetes, (5) Perception of health care services, (6) Adherence to the medication advice, (7) Adherence to the dietary advice, (8) Adherence to the physical exercise advice, (9) Adherence to the other life-style modification measures advice, (10) Cost information about the use of out-patient services (in the last 3 months), (11) Cost information about the use of in-patient services (in the last 3 months), (12) Anthropometric and bio-medical information; these measurements were collected from the patient’s prescription and diabetic guidebook. The interviews were conducted in Bangla. Each interview lasted about 45 to 50 min and took place at the BUHS hospital, Dhaka, Bangladesh.

To ensure a complete and accurate data collection, the interviewers were strongly monitored through a random check of data collection procedure. The completeness and coherence of information collected were checked during and after the data collection by checking systematically for missing values, incorrect, inappropriate and/or inconsistent entries, similar questions at different points of the interview, systematic patterns, and errors.

### The m-health intervention

There were two types of services offered under this project which were only received by the interervention group participants. The control group participants were only getting the regular hospital services. First, a patient reminder system through interactive voice calls to support the patients following the recommendations received at hospital for medication, diet, physical exercise, hospital visits, and other lifestyle modification measures (e.g. stop using tobacco, betel nuts, etc.). The voice call interaction was personalized according to the information collected during the baseline survey about the patients, their disease conditions (stage, related complications if any and other co-morbidities, and blood glucose control level) and treatments (prescribed medicine/s inlcuding name, dose, frequency, oral or insulin and other advices). Patients received a call over their mobile phone every 10 days, except Fridays and other national holidays. In case, a patient did not answer the call, it was repeated three times on the same day at one-hour interval. If it was not convenient for a patient to talk, the call was repeated at a convenient time for the patient on the same day. If a patient was still not reachable, s/he was called again within the next 3 days. A call was considered successful if a full conversation with the patient was made. The average duration of each call was 10 min. A register was maintained to record all the voice call communications. Patients also received a reminder call one or two days before their scheduled hospital visits. Second, a 24/7 call center service. Patients could access a physician to get health-related information and suggestions. The BIHS hospital was responsible for the patient reminder and the Telemedicine Reference Center Ltd. (TRCL) & e-Health Solutions was responsible for the call center. Services were provided free of cost during the intervention period. The information regarding the patients who called from the intervention group was directly received from the call center.

### Data analysis

Adherence was measured according to the following criteria: *1) Adherenceto medication intake*: a patient was considered adherent if s/he had taken the medication(s) according to the physician’s advice for dose, frequency, route of administration and duration of the treatment and without having missed any intake; *2) Adherence to lifestyle behavioral changes*: a) Diet: a patient was considered adherent if the calorie intake (carbohydrate CHO, protein, fat, and total Kilocalorie) was within a 10% margin (positive or negative) of the recommended value according to the Bangladesh Diabetes association Guide Book-BADAS patient book. Vegetable and fruit intake practice was analyzed according to recommendation, b) Physical exercise: a patient was considered adherent if s/he was walking at least 30-min per day for at least 5 days or 150 min in a week (according to WHO recommendation), c) Cessation of smoke or smokeless tobacco and betel nut: a patient was considered adherent if s/he stopped using smoke or smokeless tobacco and betel nut (according to physician advice); 3*) Biomedical outcomes*: a patient was considered at a controlled blood glucose level if the fasting blood glucose level was < 7.0 mmol/L, and the blood glucose level 2 h after breakfast was < 11.1 mmol/L (according to WHO reference value).

The effects of the m-Health intervention were analyzed by comparing the baseline and the endline estimates as well as the intervention and the control group estimates. The analysis was conducted using STATA IC 11 and considered all results with *p* values < 0.05 as being statistically significant. A multivariable logistic regression model was used to assess the association of potential factors such as age, sex, and other factors on a different dimension of adherence: total calorie intake, physical exercise, avoidance of tobacco and betel nut, and fasting blood glucose control. All models were adjusted for adherence at baseline and sex. All other behavioral and demographic variables were only kept in the final model when the reported *p*-value was < 0.05.

## Results

A total of 320 patients at baseline (160 from each group) and 273 patients at endline (142 patients from the intervention and 131 patients from the control group) were interviewed. The remaining 47 patients (18 from the intervention and 29 from the control group) could not be interviewed due to sickness, hospitalization, staying outside Dhaka during the interview period, switched off mobile phones (mainly control group), a refusal to being interviewed (mainly control group), and death.

### Sample characteristics

The mean age was 53 and 51 years in the intervention and the control group respectively. The majority of the patients were female (around 75% in both groups), which was consistent with the hospital record. More than half of the patients had finished secondary school (around 53% in both groups). Around 70% of the patients from both groups were housewives. The patients were mainly from the nuclear families (75% in both groups), and the number of family members averaged four. About 55% of the patients from both groups reported to have a monthly family income between 117 and 465 Euros (Table 1).

### Information about diseases and hospital services

The average number of years living with diabetes was seven years in the interervention and six years in the control group. In both groups, 37% of patients had a positive family history of diabetes. More than half of the patients received services at BIHS hospital for more than three years (64 and 56% in the intervention and the control group, respectively). Waiting time was less than 30 min for physician consultations and laboratory tests. The information of this section were collected only during the baseline survey.

### Adherence to medication intake and hospital visit practice

Self-reported adherence to medication intake was more than 90% in both groups at baseline and endline. Some patients reported omitting medications, physician consultations, and laboratory tests time to time due to financial problems. The omitting of medication, laboratory test and physician consultation increased between baseline and endline, both in the intervention and the control group. But the differences were not statistically significant (Intervention group: *p* = 0.102, *p* = 0.14, and *p* = 0.08 respectively; Control group: *p* = 0.25, *p* = 0.97 and *p* = 0.84 respectively) (Fig. 2).

### Adherence to dietary practice

Adherence to the recommended amount of carbohydrate and total kilocalorie intake per day improved significantly between baseline and endline (*p* = < 0.001 in both cases) in the intervention group, but not for protein and fat intake (*p* = 0.072 and *p* = 0.158 respectively). In the control group, no improvement was found in carbohydrate, protein, fat or total kilocalorie intake per day (*p* = 0.409, *p* = 0.56, *p* = 0.162 and *p* = 0.187 respectively). In both groups (at baseline and endline), the non-adherent patients reported taking a lower amount of calories per day than recommended. All patients in both groups adhered to vegetable intake recommendations at baseline and endline. Fruit intake practice increased significantly in the intervention group (*p* = < 0.001), but not in the control group (*p* = 0.260) (Fig. 3).

### Adherence to physical exercise practice

No patient reported doing moderate to vigorous intensive activity. Both groups reported having sedentary behavior, spending an average of four hours per day in a sitting position. Sedentary behavior was defined as spending time sitting at work or home, or spending time traveling in a car, bus, train, reading, playing cards or watching television. It did not include time spent sleeping. The mean number of days of exercise per week increased in the intervention group between baseline and endline (baseline: 4.6 days and endline: 6.13 days; *p* = < 0.001), but not the mean hours of exercise per day (baseline: 0.45 h and endline: 0.52 h; *p* = 0.118). In the control group, no improvement was reported either in the mean number of days of exercise per week (baseline: 5.08 days and endline: 5.28 days; *p* = 0.538) or in the mean number of hours of exercise per day (baseline: 0.57 h and endline: 0.45 h; *p* = 0.027). The preferred time for physical exercise was before breakfast in both groups (around 78%) (Table 2).

### Adherence to tobacco control practice

The use of smokeless tobacco, chewing tobacco (e.g., *Jorda*, *Gul*), and betel nut decreased three times between baseline and endline in the intervention group (from 24 to 8%; *p* = < 0.001), but increased slightly in the control group (from 28 to 32%; *p* = 0.467). The use of smoke tobacco or cigarettes decreased in both groups between baseline and endline, but this was not statistically significant (intervention group: from 2.5 to 1.4%; *p* = 0.499 and control group: from 4.4 to 2.3%; *p* = 0.333).

### Adherence to blood glucose control

The fasting blood glucose level and the blood glucose level 2 h after breakfast improved significantly in the intervention group (*p* = < 0.001 in both cases). In the control group, the blood glucose 2 h after breakfast decreased significantly (*p* = 0.035), but stayed above the normal reference value. No significant improvement was noticed in the fasting blood glucose level in the control group (*p* = 0.256) (Table 3). 

### Availing 24/7 call center services

An average of five calls per month were received by the call center from the intervention group participants duirng the one year intervention period. The advices requested were mainly nutritional, general and DM related health concerns e.g. high and low blood glucose level, and other specific health concerns e.g. heart and kindney problem, high blood pressure etc. The participants were suggested to follow their nutritional recommendations as written in their “Patient Book”. Advices on general health concerns were also given by the call center physicians. The participants were highly recommended to consult their physicians face-to-face in case of DM and other specific organ related problems.

### Factors associated with adherence

Patients from the intervention group were more likely to adhere to the recommendations on total calorie intake (OR = 4, 95% CI 2.34, 6.83, *p* < 0.001), to walking five or more days in a week (OR = 3.07, 95% CI 1.52, 6.19, *p* = 0.002), and to smokeless tobacco and betel nut cessation (OR = 4.75, 95% CI 2.16, 10.45, *p* < 0.001) than patients from the control group. Those who already adhered to walking five or more days in a week and avoided smoke or smokeless tobacco and betel nut at baseline were more likely to adhere at endline.

Male patients were more likely to adhere to dietary practice (calorie intake) than female patients. No sex difference for smokeless tobacco and betel nut intake and physical exercise practice was found. Patients older than 60 years were less likely to adhere to walking five or more days in a week than patients between 25 to 40 years. Patients with secondary or higher secondary education were more likely to adhere to the abstinence of smokeless tobacco and betel nut than patients with up to primary level education. Nuclear families, patient were more likely to adhere to smokeless tobacco and betel nut cessation than patients living in extended families. Patients with controlled fasting blood glucose were more likely to adhere to smokeless tobacco and betel nut cessation (Table 4).

## Discussion

Self-reported adherence to medication intake and to hospital visits (physician consultations and laboratory tests) was good in both groups. However, some patients reported omitting medications and hospital visits due to financial problems. The cost of treatment is a risk factor for an uncontrolled blood glucose level. Two studies conducted in Bangladesh and Turkey suggested that the diagnosis, treatment, and control rates of diabetes considerably varied by SES of the patients [31, 32]. An almost non-existent public health insurance system and a huge OOP (out of 67% OOP, 62% are contributing to purchasing medications and physician consultations) make health services unaffordable for a large group of population in Bangladesh [8]. Therefore, there is a need to implement an equitable health financing system that would allow better access to health care and protect patients from catastrophic health care expenditures [33]. The WHO suggested framework, which includes rational use of essential medications at affordable prices (insulin needs to be included), sustainable financing through public and private funding, and a reliable and functional health and supply system, should also be implemented [34, 35].

The intervention had a positive impact on adherence to dietary recommendations in this study. According to a systematic review, 70% of the identified e-Health and m-Health interventions were effective in improving dietary practices in developing countries [36]. Financial constraints were not found to be a reason for non-adherence for this in general in this study. Most of the patients who were non-adherent reported taking a lower quantity of food than recommended. This is particularly true for protein and fat intake, which could be due to the higher price of these products compared to carbohydrate products. Financial problems were reported as the main reason for dietary non-adherence in other studies [37, 38]. Among the suggestions given in the reminder calls, patients were advised to eat vegetable proteins (e.g. lentils, which is common in Bangladesh) in case of financial constraint. Male patients were more likely to adhere to dietary recommendations than women in this study, which is found consistent with a study conducted in Nepal [37]. The opposite result was found in a study conducted in Nigeria [39]. According to Zanetti et al. (2015), the association between sex and diet is not conclusive, due to differences in dietary habits and the complexity of the variables involved in adherence to dietary recommendations [40]. Therefore, continuous monitoring of the patients with a clear and attainable dietary guideline is essential. M-Health could be a good tool to ensure monitoring, allowing patients to share their successful experiences and discuss how they faced the barriers to adherence. The involvement of family members is also a key to changing dietary habits.

The impact on adherence to physical exercise was also found positive in this study. A systematic review results showed that e-& m-Health interventions could be effective in improving physical exercise practice [36]. The older patients were less likely to adhere than the younger ones in this study. Physical impairments and aging were found connected to non-adherence to physical activities or exercises in two studies conducted in Turkey and Serbia [32, 41]. A lack of information on the benefits of exercise and the believes that exercise could worsen their condition are reasons for non-adherence to exercise recommendations as well [38]. Therefore, an m-Health intervention could play an important role in motivating patients, especially the elderlies, by counseling them with proper information about the benefits of exercise and advising them to adapt it acccording to their age and physical condition.

The intervention also showed a positive effect on tobacco and betel nut cessation in this study. The habit of chewing betel nut and the use of tobacco (smoke or smokeless) is reported as a risk factor for diabetes [42, 43]. No sex difference on the practice was found in this study, though adherence was reported to be higher for women than men in other studies conducted in Bangladesh [44, 45]. The educated patients were more likely to adhere in this study; this may be because education increases individual awareness of health prevention measures [32]. Patients living in nuclear families in this study were more likely to adhere than those living in extended families. Betel nut chewing is a family tradition and part of social interaction in many countries of Asia including Bangladesh [42]. The users are influenced by older family members, so it is probably easier to stop when familial influence is not present, like in nuclear families [44].

The overall adherence to diabetes management (medication, diet, physical exercise, other lifestyle modification measures) has a direct effect on blood glucose control. The intervention group showed a significant improvement in the control of blood glucose, which was not the case in the control group. This result is found consistent to two systematic reviews [36, 46]. This bio-medical outcome also confirmed the self-reported information collected in this survey on different adherence dimensions.

At baseline, relatively high adherence to medication intake and very low adherence to other lifestyle measures were observed in both groups. One hypothesis is that physicians place more emphasis on medication intake and the patients might have less awareness in general about lifestyle modification measures; which is also observed in other studies conducted in Bangladesh and India [47, 48]. Several m-Health projects have been implemented in Bnagladesh, but this project differs as individually tailored interactive voice calls in local language was used considering the older age group patients and the low literacy rate in the country (62.5% population has no formal education) [18]. The communication was personally tailored to the needs of the patients according to the information given in the baseline survey. This provided the patients a feeling of being specially taken care of, which may have increased their motivation and feeling of responsibility to follow their treatment advices and stay adherent. One review article and two other interventional studies conducted in the USA and Malawi stated that voice call was preferred by the patients due to the personalized information [49–51]. It has also been reported by other studies that the tools work well when they are customized, and when the language and the content of the tools are highly relevant to the individual patient [50–52].

The patients enrolled in this study had access to a 24/7 call center free of cost, but very few patients made use of this service during the intervention since they did not perceive the need for it. But in case of buying the services, the cost of the calls is still high for most of the people in Bangladesh [19]. The suggested option is to offer free or subsidized call rates within the m-Health service provisions [53]. M-Health (voice call and/or SMS) and call center services through improving adherence and thereby treatment outcomes (including slowing down the complications), could help to reduce the consultations for advises or consultations for complications. This would in turn reduce some costs for the patients, removing transportation cost, and cutting the indirect cost of time off from work either due to coming to or waiting at the health facility, both for the patients and the attendants. This way at the provider level, m-Health could help to remove the additional load of unnecessary patient visits [54]. Therefore, considering the health system problems of Bangladesh and the encouraging results of m-Health interventions, it would be of great benefit to add m-Health services to the regular medical services. A public-private partnership (PPP) with non-governmental organizations (NGOs) and telecommunications companies could be considered to provide free or subsidized m-Health services to the population, especially to those with lower SES, addressing the issue of accessibility, availability, and affordability.

## Limitations

This study has several limitations. First, the patients were recruited from a tertiary care hospital (BIHS) in Dhaka, Bangladesh. Therefore, the patients were unlikely to represent all patients with DM 2 in the country, which influences the generalizability of the results. Second, female participation was significantly higher in both groups, which was found consistent with the hospital records. Third, a total of 47 participants could not be interviewed at endline. But these individuals were not significantly different from the study population. Fourth, adherence was self-reported, which may lead to an over-report of adherence (social desirability bias). To overcome this, direct measures of clinical outcomes (blood glucose values) were added.

## Conclusions

The study showed a positive impact of the m-Health intervention on patients´ adherence particularly for diet, physical exercise, cessation of tobacco and betel nut, and control of blood glucose levels. Financial constraints were found to be the main reason for omitting medications and hospital visits and were also linked to uncontrolled blood glucose level. An almost non-existent public health insurance system and a huge OOP threatened the treatment at the patient level. A public health insurance system and the enlisting of NCD medications to the essential medicine list would help reduce inequity and financial constraints. The individually tailored interactive voice calls complemented the information given by physicians to patients during the consultations, highlighting in particular the broader aspects of the management of diabetes and the importance of not focusing solely on medications. This has proven effective in increasing adherence and in giving adequate support to the patients to better manage their disease.

## Data Availability

Data will be available on request. The first author is the responsible person to contact for data access.

## Abbreviations

BADAS: Bangladesh Diabetes Association
BIHS: Bangladesh Institute of Health Sciences
BUHS: Bangladesh University of Health Sciences
DALY: Disability-adjusted life years
DM 2: Diabetes Mellitus Type 2
CHO: Carbohydrate
HIGH: Heidelberg Institute of Global Health
LMIC: Low-and middle-income country
m-Health: Mobile health
NCD: Non-communicable disease
NGO: Non-government organization
NIPORT: National Institute of Population Research and Training
OOP: Out-of-pocket payment
PPP: Public-private partnership
RCT: Randomized Control Trial
SMS: Short message service
SES: Socio-economic status
TRCL: Telemedicine Reference Center Ltd.
USA: United States of America
WHO: World Health Organization
YLL: Years of life lost
